# Structural Diversity of Six Coordination Polymers Based on the Designed X-Shaped Ligand 1,1,1,1-Tetrakis[(3-pyridiniourea)methyl]methane

**DOI:** 10.3390/molecules23092292

**Published:** 2018-09-07

**Authors:** Qi-Long Zhang, Qing Yu, Hai-Fang Xie, Bo Tu, Hong Xu, Ya-Li Huang, Xiao-Sheng Yang

**Affiliations:** 1 State Key Laboratory of Functions and Applications of Medicinal Plants, Guizhou Medical University, Guiyang 550014, China; gzcnp@sina.cn; 2 School of Basic Medical Science, Guizhou Medical University, Guiyang 550004, China; yq518328@126.com (Q.Y.); x1352316768@126.com (H.-F.X.); xuhong@gmc.edu.cn (H.X.); ylh6401@gmc.edu.cn (Y.-L.H.)

**Keywords:** 1,1,1,1-tetrakis[(3-pyridiniourea)methyl]methane, coordination polymers, single crystal structures

## Abstract

In this study, six coordination polymers (CPs), {[Ag_2_(L)(CF_3_SO_3_)]·CF_3_SO_3_·2H_2_O·DMF}_n_ (**1**), {[Ag(L)]·SbF_6_·4DMF·H_2_O}_n_ (**2**), {[Zn(L)_0.5_(I)_2_]·3.75H_2_O}_n_ (**3**), {[Cd_2_(L)(I)_4_(H_2_O)(DMF)]·4H_2_O·3DMF}_n_ (**4**), {[Hg_2_(L)(I)_4_]·H_2_O·4DMF}_n_ (**5**) and {[Hg_2_(L)(Cl)_4_]·2H_2_O·3DMF}_n_ (**6**), were obtained based on the designed X-shaped urea-based ligand. X-ray single crystal diffraction analysis revealed that complex **1** displayed a 3D (3,4)-connected {6·8^2^}{6^4^·8^2^}-**tcj** net. Complex **2** featured a 2D 4-connected {4^3^·6^3^} sheet. Complexes **3** and **5** exhibited a 1D polymeric loop chain. Complex **4** displayed a 1D polymeric fishbone chain. Complex **6** showed a 2D 4-connected {4^4^·6^2^}-**sql** sheet. Structural comparison revealed that not only the metal ions, but also the anions played crucial roles in the control of final structures.

## 1. Introduction

Coordination polymers (CPs), which act as fluorescence probes, photoelectric sensors, zeolites, drug delivery systems, catalysts, molecular switches, and devices used in the chemical, industrial and medical fields, have attracted considerable attention due to their fascinating structures and potential applications [1,2,3]. Although research on their coordination chemistry is focused on building functional CPs, the design and assembly of CPs are of interest as their applications are determined by their properties, which are essentially determined by their structures [4,5].

Usually, CPs are constructed from organic ligands and inorganic metal building species under suitable conditions [6,7]. For example, 1D CPs are considered to have the least structural framework, in particular, metal coordination-based noncovalent interactions between 1D infinite chains can lead to interesting higher-dimensional architectures [8,9]. These include linear, helical, rotaxane, ladder and ribbon/tape structures, which have been successfully demonstrated in recently years [10,11]. Of these structures, tape-like or ribbon polymers are of special interest, not only due to their beautiful architecture, but also their pores or cavities which facilitate the encapsulation of guest molecules, such as in gas adsorption [12]. However, difficulties have arisen due to the need for a complementary ligand/cation pair, which is capable of offering reversible and mutually favorable interconnections. In practice, many factors such as the coordination modes of the metal ions, counter-anions, as well as steric bulk, solvent, metal−ligand ratio, and the pH of the system need to be considered [13]. Therefore, the development of new ligands with highly pre-organized and flexible coordination properties remain a challenge [14].

Recently, bis- and tris(pyridylurea)-based ligands have been proved to be both excellent anion receptors and building blocks for novel supramolecular CPs architectures [15,16]. For example, a number of metal–ligand supramolecular networks of oligo(pyridylurea)-based ligands have been investigated. In contrast, the networks based on tetrakis(pyridylurea)-based ligands which possess more coordination points and flexible chains, may lead to more fascinating and diverse ones, are still rare. It is believed that different coordination environments can be achieved by adjusting their conformation, length or inherent angle of the terminal coordinative groups [17,18].

**Figure 1:**
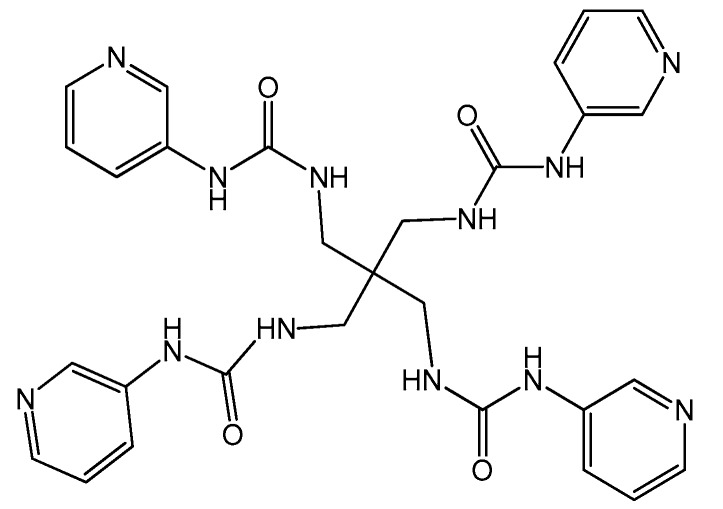
The molecular structure of the X-shaped urea-based ligand **L**.

Inspired by the abovementioned points and following our recent studies [19], we designed a novel X-shaped urea-based ligand **L** (Figure 1), reacted it with transition metal ions, and finally obtained the following six CPs with distinct structures: {[Ag_2_(L)(CF_3_SO_3_)]·CF_3_SO_3_·2H_2_O·DMF}_n_ (**1**), {[Ag(L)]·SbF_6_·4DMF·H_2_O}_n_ (**2**), {[Zn(L)_0.5_(I)_2_]·3.75H_2_O}_n_ (**3**), {[Cd_2_(L)(I)_4_(H_2_O)(DMF)]·4H_2_O· 3DMF}_n_ (**4**), {[Hg_2_(L)(I)_4_]·H_2_O·4DMF}_n_ (**5**), and {[Hg_2_(L)(Cl)_4_]·2H_2_O·3DMF}_n_ (**6**). Most interestingly, a 1D loop chain was successfully formed by the X-shaped urea-based ligand.

## 2. Results and Discussion

### 2.1. Structural Description of {[Ag_2_(L)(CF_3_SO_3_)_2_]·2H_2_O·DMF}_n_ *(**1**)*

**Figure 2:**
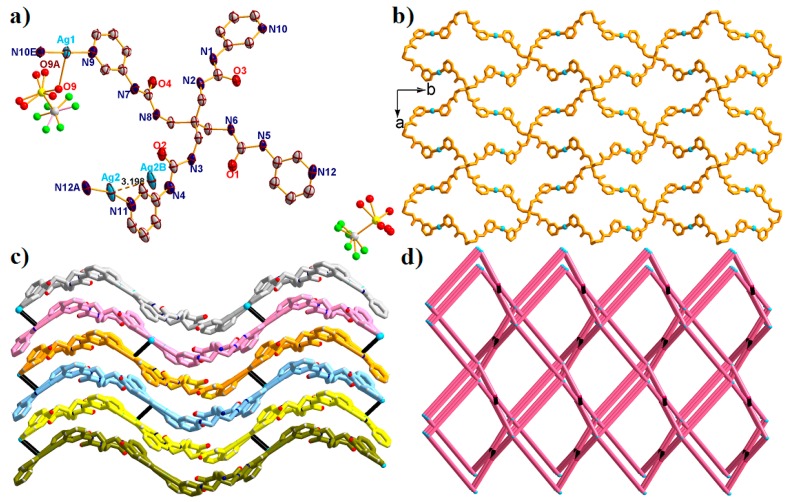
(**a**) The asymmetric unit of complex **1** with 50% probability ellipsoids. All the solvents and hydrogen atoms have been omitted for clarify. (Symmetry codes: A: 1 − *x*, −1/2 + *y*, 3/2 − *z*; B: 1 − *x*, −1 − *y*, 2 − *z*; E: −*x*, −1/2 + *y*, 1/2 − *z*). (**b**) The 2D [Ag_2_(L)]_n_ layer of **1** view along *c* direction. (**c**) The Ag···Ag interactions expanded 3D framework of **1**. (**d**) The simplified **3D** (3,4)–connected {6·8^2^}{6^4^·8^2^}-**tcj** net of **1**.

Structural analysis revealed that complex **1** crystallized in the monoclinic system, space group *P*2_1_/c. As shown in Figure 2a, the asymmetric unit consists of two crystallographically independent Ag^I^ ions, one L ligand, one coordinated CF_3_SO_3_^–^ anion, one free CF_3_SO_3_^–^ anion, two lattice water molecules and one free DMF solvent molecule. Ag(1) ion is tricoordinated with two N atoms (N9 and N10E) from two different L ligands, and one O atom (O9) from the coordinated CF_3_SO_3_^–^ anion, while Ag(2) ion is dicoordinated with two N atoms (N11 and N12A) from two different L ligands. In addition, the Ag-N bond lengths span the range of 2.110 Å–2.132 Å, and the Ag-O distance is 2.541 Å. In complex **1**, the L ligand acts as the X-shaped bridge to connect four Ag^I^ ions using the pyridine N atoms, with the τ_4_ parameter of the central C atom in the {CAg_4_} tetrahedron = 0.44(7) with *α* = 152.22° and *β* = 144.69° (τ_4_ = [360° − (*α + β*)]/141°; *α* and *β* are the two largest bond angles), a 2D wave [Ag2(L)]n sheet is successfully constructed with the nearest Ag···Ag distances being 9.290 Å (Ag1···Ag2) and 9.846 Å (Ag1···Ag2C, Symmetry code: C: −1 + *x*, *y*, −1 + *z*) (Figure 2b). It is worth noting that the neighboring [Ag_2_(L)]_n_ sheets interacted with each other through the Ag···Ag bonds with a bond length of 3.198 Å (Ag2···Ag2B). A 3D framework (Figure 2c) was finally constructed, in which the [Ag_2_(L)]_n_ sheets were arranged alternately. After omitting the lattice solvent molecules as well as the CF_3_SO_3_^–^ anions, the framework showed interesting porous nets with porosity of approximately 27.3%, calculated by PLATON [20].

From the viewpoint of topology, the final 3D structure can be simplified into an interesting (3,4)-connected tcj net with the Point symbol of {6·8^2^}{6^4^·8^2^} by denoting the L ligands, and the Ag^I^ ions as 4-connected and 3-connected nodes, respectively (Figure 2d).

### 2.2. Structural Description of {[Ag(L)]·SbF_6_·4DMF·H_2_O}_n_ *(**2**)*

**Figure 3:**
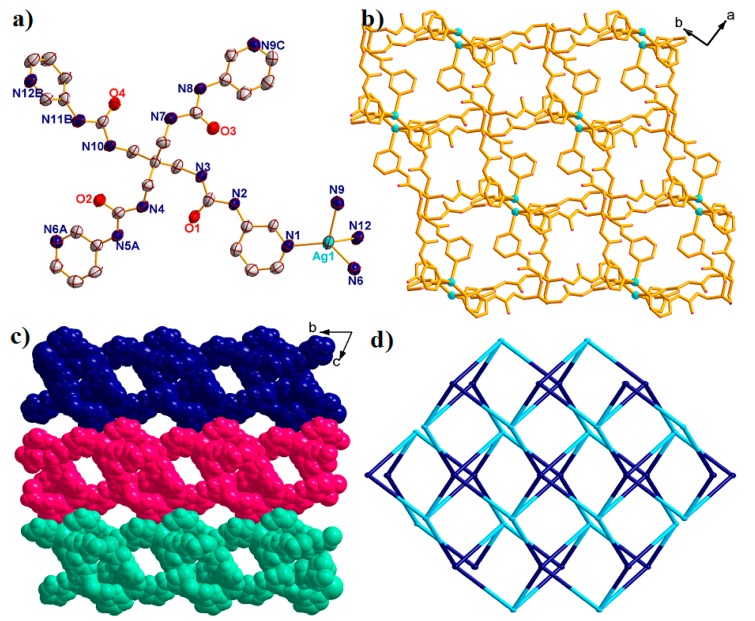
(**a**) The asymmetric unit of complex **2** with 50% probability ellipsoids. All the solvents and hydrogen atoms have been omitted for clarify. (Symmetry codes: A: *x*, 1 + *y*, *z*; B: −1 + *x*, 1 + *y*, *z*; C: 1 − *x*, −1 − *y*, 2 − *z*.). (**b**) The 2D [Ag(L)]_n_ bilayer of **2** view along *c* direction. (**c**) Space filling 3D supramolecular structure of **2** view along *a* direction. (**d**) The simplified **2D** 4-connected {4^3^·6^3^} sheet of **2**.

Structural analysis revealed that complex **2** crystallized in the triclinic space group *P-1*. The asymmetric unit consists of one Ag^I^ ion, one L ligand, one free SbF_6_^–^ anion, four free DMF molecules and one lattice water molecule (Figure 3a).

The central Ag^I^ ion is tetracoordinated with four N atoms from four different L ligands, leaving a distorted {AgN_4_} tetrahedral geometry with a τ_4_ parameter of 0.82(0). In addition, the Ag-N bond lengths span the range of 2.239 Å–2.415 Å. Different to that in complex **1**, the L ligands also act as the X-shaped linkers connecting the Ag^I^ ions using the pyridine N atoms to form a 2D [Ag(L)]_n_ bilayer with the nearest Ag···Ag distances being 10.980 Å and 14.652 Å (Figure 3b). The τ_4_ parameter of the central C atom in the {CAg_4_} tetrahedron is 0.49(7) with α = 150.38° and β = 139.48°. Interestingly, the bilayer holds a 1D channel, with an opening area of approximately 8.914 × 12.346 Å^2^ along the a direction, in which the free solvents fulfilled. The adjacent [Ag(L)]_n_ bilayer interacted with each other through hydrogen bonds, further expanding into a 3D supramolecular structure (Figure 3c). From the viewpoint of topology, the bilayer structure of complex **2** can be defined as a 4-connected sheet with the Point symbol of {4^3^·6^3^} by denoting the L ligands as well as the Ag^I^ ions both as 4-connected nodes (Figure 3d).

### 2.3. Structural Description of {[Zn(L)_0.5_(I)_2_]·3.75H_2_O}_n_ *(**3**)*

**Figure 4:**
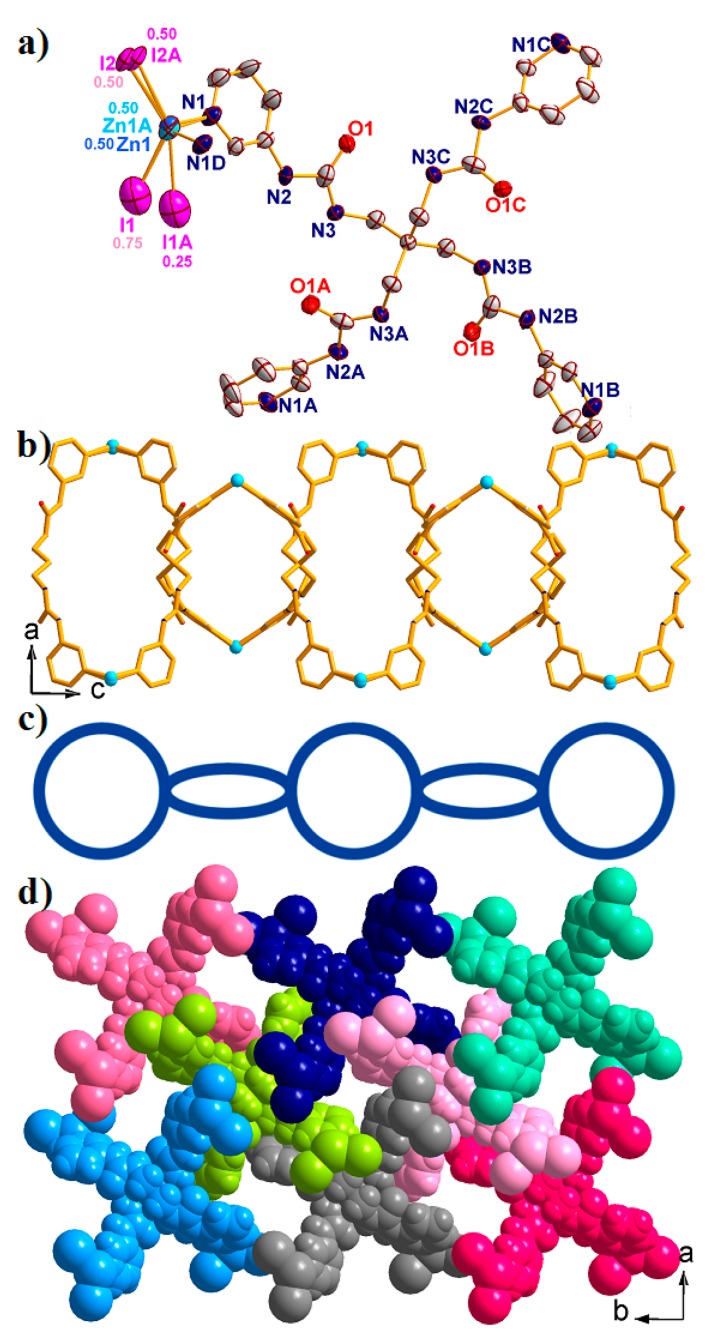
(**a**) The asymmetric unit of complex **3** with 50% probability ellipsoids. (Symmetry codes: A: 1/2 + *x*, 3/2 − *y*, 1/2 − *z*; B: 2 − *x*, 1 − *y*, *z*; C: 3/2 − *x*, −1/2 + *y*, 1/2 − *z*; D: *x*, *y*, −*z*.). (**b**) The 1D [Zn_2_(L)]_n_ loop chain of 3 view along *b* direction. (**c**) The simplified 1D loop chain structure. (**d**) Space filling 3D supramolecular structure of 3 view along *c* direction. All the solvents have been omitted for clarify.

Structural analysis revealed that complex **3** crystallized in the tetragonal space group *I*4/m. The asymmetric unit consists of one Zn^II^ ion, a half of L ligand, two coordinated I^–^ anions, and three and three-quarter lattice water molecules. As shown in Figure 4a, the central Zn^II^ ion is tetracoordinated with two N atoms from two different L ligands [Zn1-N1 = 2.019(7) Å, and Zn1-N1D = 2.013(4) Å] and two I^–^ anions [Zn1-I1 = 2.636(9) Å, and Zn1-I2 = 2.576(5) Å], leaving a distorted {ZnN_2_I_2_} tetrahedral geometry with a τ_4_ parameter of 0.88(5).

In building complex **3**, the L ligand also acts as the X-shaped linker to connect four Zn^II^ ions through pyridine N atoms, successfully forming a 1D [Zn(L)]_n_ loop chain structure with the nearest Zn···Zn distances being 14.506 Å for Zn1···Zn1C and 14.480 Å for Zn1···Zn1A (Figure 4b and Figure 4c). The τ_4_ parameter of the central C atom in the {CZn_4_} tetrahedron is 0.67(0) with *α* = *β* = 132.78°. In addition, the ellipsoidal loop structure contains two Zn^II^ ions and two half L ligands, with an opening area of 6.819 × 17.517 Å^2^. The neighboring loop chain structure further interacted with each other through hydrogen bonding, finally resulting in a 3D supramolecular structure (Figure 4d).

### 2.4. Structural Description of {[Cd_2_(L)(I)_4_(H_2_O)(DMF)]·4H_2_O·3DMF}_n_ *(**4**)*

**Figure 5:**
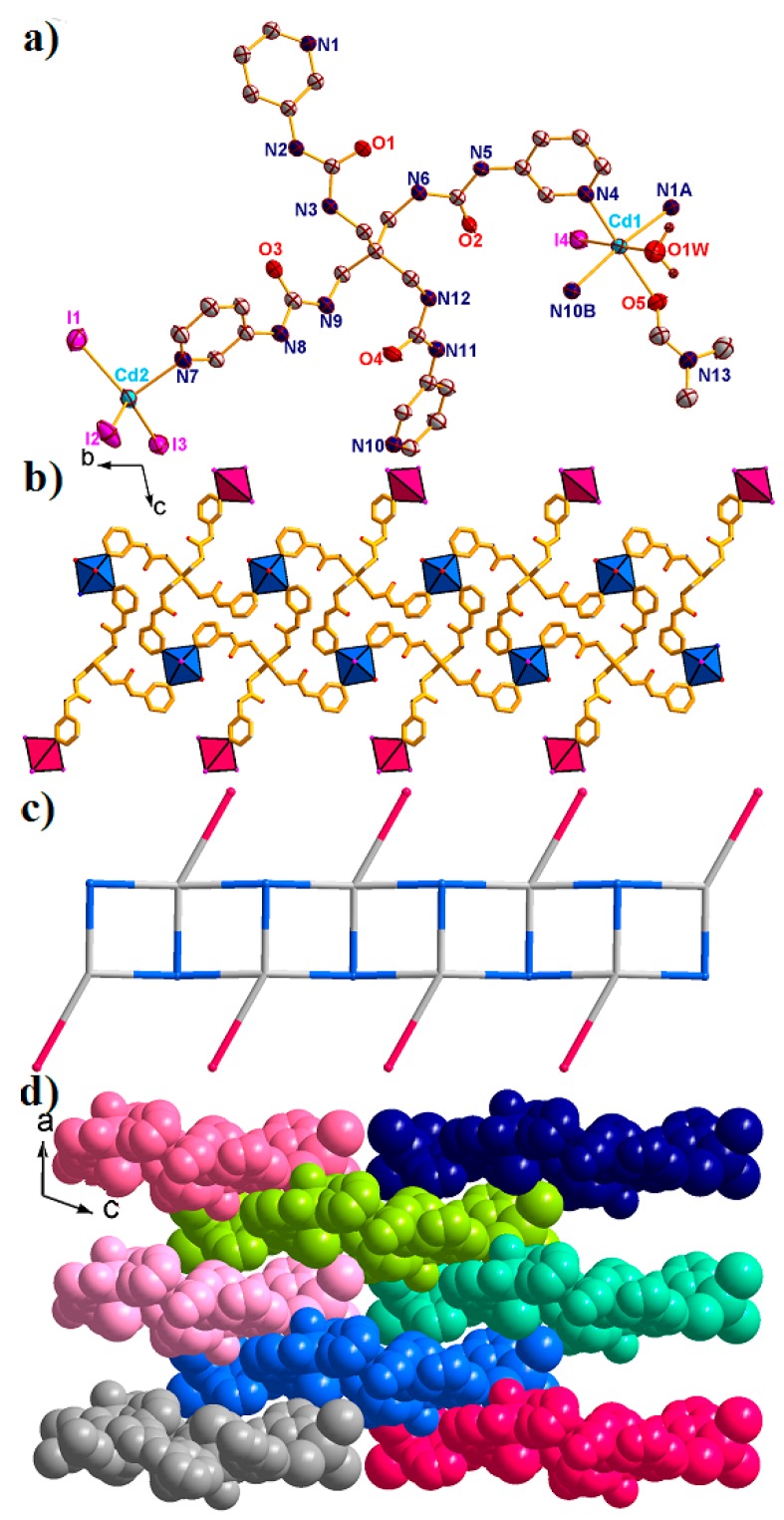
(**a**) The asymmetric unit of complex **4** with 50% probability ellipsoids. (Symmetry codes: A: 2 − *x*, 1 − *y*, −1 − *z*; B: 2 − *x*, −*y*, −1 − *z*). (**b**) The 1D [Cd_2_(L)]_n_ fishbone chain of **4** viewed along the *a* direction. (**c**) The simplified 1D fishbone chain structure. (**d**) Space filling 3D supramolecular structure of **4** viewed along the *b* direction. All solvents have been omitted for clarity.

When CdI_2_ was used to replace ZnI_2_, the obtained polymic chains changed from 1D loop chains to fishbone chains. Complex **4** crystallized in the triclinic system *P-1*. There are two Cd^II^ ions, one L ligand, four coordinated I^–^ anions, one coordinated DMF molecule, one coordinated water molecule, three free DMF molecules, and four lattice water molecules in the asymmetric unit. As shown in Figure 5a, the coordination environments of Cd^II^ ions are different to the Zn^II^ ion in complex **3**.

Cd(1) is octacoordinated by three N atoms from three L ligands, two O atoms from coordinated DMF and water molecules, and one I^–^ anion, resulting in distorted {CdN_3_O_2_I} octahedral coordination geometry. The Cd(2) located in the distorted {CdNI_3_} tetrahedral geometry, was completed by one N atom from one L ligand and three I^–^ anions, with a τ_4_ parameter of 0.88(1). In addition, the Cd-O/N bond lengths are in the range of 2.325(5)–2.406(7) Å, and the Cd–I distances span the range of 2.727(6)–2.854(9) Å, respectively. During the formation of complex **4**, each L ligand linked with three Cd1 and one Cd2 ions, resulting in a 1D [Cd_2_L]_n_ chain, in which the two nearest Cd···Cd distances were 9.279 Å for Cd2···Cd1B and 12.092 Å for Cd1···Cd1A (Figure 5b). The other coordination sites of the Cd^II^ ions are occupied by the solvent molecules and I^–^ anions, finally resulting in a 1D fishbone polymeric chain structure (Figure 5c). The τ_4_ parameter of the central C atom in the {CCd_4_} tetrahedron is 0.36(0) with α = 158.18° and β = 151.07°. The adjacent fishbone chains are further expanded into the 3D supramolecular structure through hydrogen bonding (Figure 5d).

### 2.5. Structural Description of {[Hg_2_(L)(I)_4_]·H_2_O·4DMF}_n_ *(**5**)*

**Figure 6:**
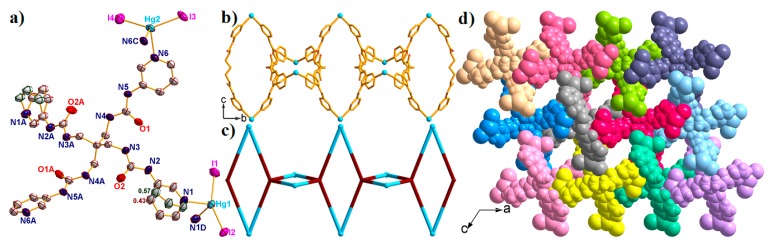
(**a**) The asymmetric unit of complex **5** with 50% probability ellipsoids. (Symmetry codes: A: −*x*, *y*, 1 − *z*; C: *x*, 2 − *y*, *z*; D: *x*, 3 − *y*, *z*). The 1D [Hg_2_L]_n_ polymeric chain (**b**) and the 1D simplified structure (**c**) viewed along the *a* direction. (**d**) Space filling 3D supramolecular structure of **5** viewed along the *b* direction. All solvents have been omitted for clarity.

Complex **5** crystallized in the monoclinic system *C*2/*m*. There are two crystallographically independent Hg^II^ ions, one L ligand, four I^–^ anions, one lattice water molecule, and four free DMF molecules in the asymmetric unit. The coordination environments of the two Hg^II^ ions are similar, both located in the distorted tetrahedral {HgN_2_I_2_} geometry, surrounded by two N atoms from two different L ligands and two I^–^ anions (Figure 6a). The τ_4_ parameters for Hg(1) and Hg(2) are 0.76(5) and 0.81(3), respectively. Moreover, the Hg-N bond lengths are 2.402(6) Å and 2.452(6) Å, and the Hg-I distances span the range of 2.641(9)–2.650(5) Å, respectively.

In complex **5**, each L ligand is linked with two Hg1 and two Hg2 ions, resulting in a 1D [Hg_2_L]_n_ loop chain, in which the two nearest Hg···Hg distances are 14.315 Å for Hg1···Hg2 and 15.665 Å for Hg1···Hg2A (Figure 6b). In addition, the I^–^ anions further occupied the other coordination sites of the Hg^II^ ions, finally resulting in a 1D loop chain structure. The τ_4_ parameter of the central C atom in the {CHg_4_} tetrahedron is 0.65(5) with *α* = 137.49° and *β* = 130.21°. When the L ligand was defined as X-shaped 4-connected nodes, the loop chain structure can be simplified into a 1D chain (Figure 6c). Through hydrogen bonding, these chains can be further expanded into a 3D porous supramolecular structure, in which the solvents occupied the channels (Figure 6d).

### 2.6. Structural Description of {[Hg_2_(L)(Cl)_4_]·2H_2_O·3DMF}_n_ *(**6**)*

**Figure 7:**
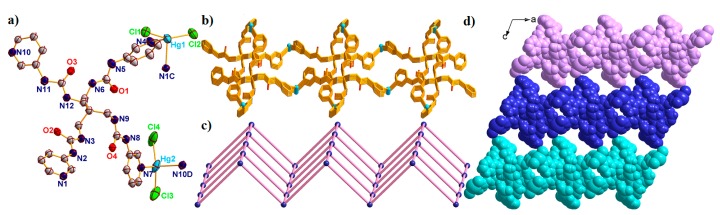
(**a**) The asymmetric unit of complex **6** with 50% probability ellipsoids. (Symmetry codes: C: 2 − *x*, −1/2 + *y*, 1 − *z*; D: −1 + *x*, *y*, *z*). The 2D [Hg_2_L]_n_ sheet. (**c**) The 2D 4-connected {4^4^·6^2^}-**sql** sheet of **6**. (**d**) Space filling 3D supramolecular structure of **6** viewed along the *b* direction. All solvents have been omitted for clarity.

X-ray single-crystal analysis revealed that complex **6** crystallized in monoclinic system, *P*2_1_ space group and the asymmetric unit consists of two Hg^II^ ions, one L ligand, four Cl^–^ anions, two lattice water molecules, and three free DMF molecules. One half of a bimb ligand lies in the independent inversion center, and a half of lattice water molecules. As shown in Figure 7a, two Hg^II^ ions display similar coordination environments, completed by two N atoms from two different L ligands and two Cl^–^ anions, both located in the distorted tetrahedral {HgN_2_Cl_2_} geometry, with the τ_4_ parameter for Hg(1) and Hg(2) at 0.73(6) and 0.75(8), respectively. In addition, the Hg-N bond lengths are in the range of 2.397(8)–2.449(3) Å, and the Hg-Cl distances span the range of 2.314(6)–2.330(6) Å, respectively.

In the assembly of complex **6**, each L ligand is linked with two Hg1 and two Hg2 ions, resulting in a 2D [Hg_2_L]_n_ bilayer, in which the two nearest Hg···Hg distances are 13.019 Å for Hg1···Hg2 and 14.856 Å for Hg1···Hg2B (Symmetry code: B: 1 + *x*, *y*, *z*) (Figure 7b). The Cl^–^ anions further occupied the other coordination sites of the Hg^II^ ions, finally resulting in a 2D sheet. The τ_4_ parameter of the central C atom in the {CHg_4_} tetrahedron is 0.78(1) with *α* = 130.11° and *β* = 119.71°. When the L ligand was defined as X-shaped 4-connected nodes, the bilayer can be simplified into a 2D 4-connected {4^4^·6^2^}-**sql** sheet (Figure 7c). In addition, these bilayers interacted with the adjacent bilayers through hydrogen bonding, and finally expanded into a 3D supramolecular structure (Figure 7d).

### 2.7. Structural Comparison

**Figure 8:**
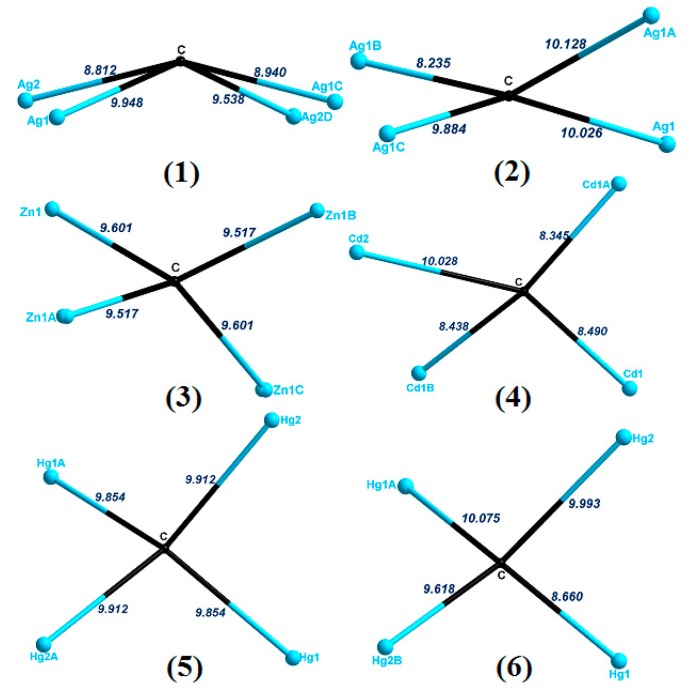
The simplified **L** connected the metal ions in complexes **1**–**6**.

As shown in Table 1 and Figure 8, although the L ligand acts as X-shaped 4-connected linkers in all six complexes, the obtained [ML_x_]_n_ motifs are distinct from the 2D [Ag_2_L]_n_ sheet, 2D [AgL]_n_ sheet, 1D [M_2_L]_n_ loop chain, 1D [Cd_2_L]_n_ fishbone chain, and the 2D [Hg_2_L]_n_ bilayer, which can be mainly attributed to the flexibility of the **L** ligand (τ_4_ parameter of the central C atom in the {CM_4_} tetrahedron was introduced to indicate the degree of flexibility), the anions, and the coordination preferences of metal ions: (i) the τ_4_ parameter of the central C atom of the {CM_4_} tetrahedron transformed from 0.36(0) for **4** to 0.78(1) for **6**, indicating that the flexible backbone of the ligand expanded directions, which are distinct when connecting the metal ions by rotating, twisting, folding, or bending; (ii) the coordination sites of the Ag^I^ ions can be 2 and 3 in complex **1**, and **4** in complex **2**, the Zn^II^ ions in complex **3** and Hg^II^ ions in complex **5**/**6** tend to adopt tetrahedral geometry, while the Cd^II^ ion was located in distorted octahedral coordination geometry in complex **4**; (iii) the different anions also play important roles in determining the structural diversity, and the reaction conditions are the same except the metal salts. The anions can coordinate with the metal ions or just act as the charge balance. By comparing complexes **1**/**2** and **5**/**6**, we noted that different anions also have preferences in controlling the structures. It is noteworthy that the Ag···Ag connections in complex **1** further expanded the final structure into a 3D framework. In addition, other motifs were further expanded into the 3D supramolecular structures through hydrogen bond. Overall, by adjusting the starting reaction salts, six CPs were obtained with structures ranging from a 1D polymeric fishbone chain (**4**), 1D polymeric loop chain (**3** and **5**), 2D 4-connected {4^4^·6^2^}-**sql** sheet (**6**), 2D 4-connected {4^3^·6^3^}sheet (**2**), to a 3D (3,4)-connected {6·8^2^}{6^4^·8^2^}-**tcj** net (**1**).

### 2.8. Powder X-ray Diffraction Analyses (PXRD)

In order to check the phase purity of these complexes, the PXRD patterns of the title series of complexes were confirmed at room temperature. As shown in Supplementary Materials Figure S1, the peak positions of the simulated and experimental PXRD patterns are in agreement with each other, demonstrating the good phase purity of the complexes. The dissimilarities in intensity may be due to the preferred orientation of the crystalline powder samples.

## 3. Experimental Section

### 3.1.Materials and Methods

All chemical reagents were obtained from commercial sources and used without further purification. IR spectra were measured on a NEXUS 670 FTIR spectrometer in the range of 400–4000 cm^–1^. Elemental analyses were carried out on a CE instruments EA 1110 elemental analyzer. X–ray powder diffractions of the title series of complexes were measured on a Panalytical X–Pert pro diffractometer with Cu–Kα radiation.

### 3.2. Synthesis of the Ligand and Complexes

#### 3.2.1. Design of 1,1,1,1-tetrakis[(3-pyridiniourea)methyl]methane (**L**)

**Figure 9:**
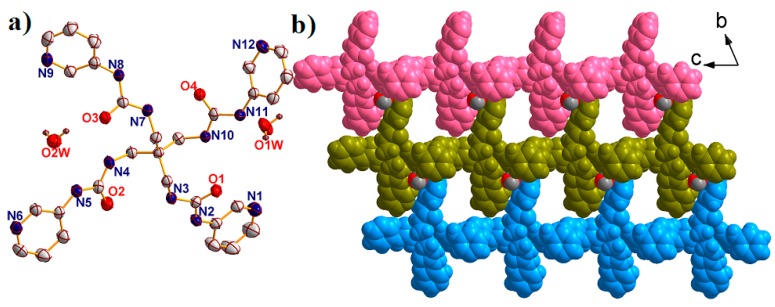
(**a**) The asymmetric unit of **L·2H_2_O**. (**b**) The 3D packing diagram of **L·2H_2_O** view along *a* direction.

Pentaerythrityltetramine (1 mmol, 0.132 g) was dissolved in ethanol (10 mL) and then added dropwise into a toluene solution (50 mL) of 3-isocyanatopyridine (4 mmol, 0.592 g). The mixture was refluxed for 2 h, then the resulting clear colorless mixture was slowly cooled to room temperature, the white precipitate was filtered off, washed with toluene and diethyl ether twice, and recrystallized from ethanol. The yield was 68%. Anal. (%) calcd. for C_29_N_12_O_4_H_32_: C, 56.85; H, 5.26; N, 27.44. Found: C, 56.63; H, 5.32; N, 27.71. IR (KBr pellet, cm^−1^): 3337 (s), 3058 (w), 2938 (w), 1677 (s), 1546 (vs), 1519 (vs), 1481 (s), 1420 (s), 1306 (s), 1235 (s), 1142 (w), 1104 (w), 1033 (w), 869 (w), 798 (m), 705 (m), 629 (w). The^1^H-NMR spectrum (Figure S1) also proved the structure. The asymmetric unit as well as the 3D packing diagram of the L·2H_2_O species is given in Figure 9.

#### 3.2.2. Preparation of {[Ag_2_(L)(CF_3_SO_3_)]·CF_3_SO_3_·2H_2_O·DMF}_n_ (**1**)

AgCF_3_SO_3_ (0.454 g, 0.4 mmol) in ethanol (20 mL) was added dropwise with stirring into a solution of **L** (0.226 g, 0.1 mmol) in a mixed solution of ethanol/DMF (20 mL) and stiiring was continued at room temperature for several days. Colourless block crystals suitable for X-ray diffraction studies were obtained by slow diffusion of the filtrate after 5 days. The crystals were filtered off, washed with the mother solution and diethyl ether, and dried in air to give 1 in 55% yield based on L. Anal. (%) calc. for C_34_H_43_Ag_2_F_6_N_13_O_13_S_2_: C, 33.05; H, 3.51; N, 17.74. Found: C, 33.01; H, 3.56; N, 17.82. IR (KBr pellet, cm^−1^): 3370 (s), 2934 (w), 1665 (s), 1603 (m), 1548 (vs), 1420(m), 1262 (vs), 1167 (m), 1104 (w), 1037 (m), 880 (w), 805 (m), 702 (m), 641(m), 573 (w), 519 (w).

#### 3.2.3. Preparation of {[Ag(L)]·SbF_6_·4DMF·H_2_O}_n_ (**2**)

The same synthetic procedure as for **1** was used, except that AgCF_3_SO_3_ was replaced by AgSbF_6_, giving colorless block crystals. The precipitate that formed was collected by filtration, and dried at room temperature to give 2 in 43% yield based on **L**. Anal. (%) calc. for C_79_H_117_Ag_2_F_12_N_31_O_17_Sb_2_: C, 38.57; H, 4.79; N, 17.65. Found: C, 38.52, H, 4.83; N, 17.70. IR (KBr pellet, cm^−1^): 3352 (s), 3085 (w), 2933 (w), 1682 (s), 1600 (m), 1546 (vs), 1420 (m), 1300 (s), 1235 (s), 1109 (w), 1037 (m), 874 (w), 805 (m), 705 (m), 629 (m).

#### 3.2.4. Preparation of {[Zn(L)_0.5_(I)_2_]·3.75H_2_O}_n_ (**3**)

The same synthetic procedure as for **1** was used, except that AgCF_3_SO_3_ was replaced by ZnI_2_, giving colorless block crystals. The precipitate that formed was collected by filtration, and dried at room temperature to give 3 in 36% yield based on **L**. Anal. (%) calc. for C_58_H_94_N_24_O_23_Zn_4_I_8_: C, 25.13; H, 3.42; N, 12.13. Found: C, 24.97; H, 3.46; N, 12.17. IR (KBr pellet, cm^−1^): 3374 (s), 3074 (w), 2938 (w), 2861 (w), 1682 (s), 1606 (s), 1557 (vs), 1503 (vs), 1486 (s), 1420 (m), 1306 (s), 1241 (s), 1104 (w), 1060 (m), 880 (w), 805 (m), 749 (m), 694 (m), 651 (m), 564 (w), 421 (w).

#### 3.2.5. Preparation of {[Cd_2_(L)(I)_4_(H_2_O)(DMF)]·4H_2_O·3DMF}_n_ (**4**)

The same synthetic procedure as for **1** was used, except that AgCF_3_SO_3_ was replaced by CdI_2_, giving colorless block crystals. The precipitate that formed was collected by filtration, and dried at room temperature to give 4 in 39% yield based on **L**. Anal. (%) calc. for C_41_H_70_Cd_2_I_4_N_16_O_13_: C, 28.51; H, 4.08; N, 12.97. Found: C, 28.45; H, 4.12; N, 13.02. IR (KBr pellet, cm^−1^): 3358 (s), 3189 (m), 3129 (m), 2933 (w), 1655 (vs), 1595 (m), 1546 (vs), 1481 (s), 1420 (s), 1300 (s), 1235 (s), 1109 (m), 1055 (w), 874 (w), 809 (w), 700 (m), 667 (m), 416 (w).

#### 3.2.6. Preparation of {[Hg_2_(L)(I)_4_]·H_2_O·4DMF}_n_ (**5**)

The same synthetic procedure as for **1** was used, except that AgCF_3_SO_3_ was replaced by HgI_2_, giving colorless block crystals. The precipitate that formed was collected by filtration, and dried at room temperature to give 5 in 28% yield based on **L**. Anal. (%) calc. for C_41_H_62_Hg_2_I_4_N_1__6_O_9_: C, 26.88; H, 3.41; N, 12.23. Found: C, 26.82; H, 3.45; N, 12.28. IR (KBr pellet, cm^−1^): 3374 (s), 2933 (w), 1677 (s), 1612 (m), 1546 (vs), 1486 (m), 1420 (m), 1306 (s), 1235 (m), 1109 (w), 1050 (w), 874 (w), 804 (m), 749 (m), 694 (w), 646 (w).

#### 3.2.7. Preparation of {[Hg_2_(L)(Cl)_4_]·2H_2_O·3DMF}_n_ (**6**)

The same synthetic procedure as for **1** was used, except that AgCF_3_SO_3_ was replaced by HgCl_2_, giving colorless block crystals. The precipitate that formed was collected by filtration, and dried at room temperature to give 6 in 25% yield based on **L**. Anal. (%) calc. For C_38_H_58_Cl_4_Hg_2_N_15_O_9_: C, 32.32; H, 4.14; N, 14.88. Found: C, 32.28; H, 4.18; N, 14.92. IR (KBr pellet, cm^−1^): 3364 (s), 3091 (w), 2927 (w), 1666 (s), 1612 (m), 1546 (vs), 1481 (m), 1420(m), 1300 (m), 1235 (m), 1109 (w), 1050 (w), 880 (w), 804 (m), 743 (w), 700 (w), 629 (w), 416 (w).

### 3.3. X–ray Crystallography

Intensity data collection was carried out on a Siemens SMART diffractometer equipped with a CCD detector using Mo-Kα monochromatized radiation (*λ* = 0.71073 Å) at 296 (2) K. The absorption correction was based on multiple and symmetry–equivalent reflections in the data set using the SADABS program. The structures were solved by direct methods and refined by full–matrix least–squares using the SHELXTL package [20,21].

All non–hydrogen atoms were refined anisotropically. Hydrogen atoms except those on water molecules were generated geometrically with fixed isotropic thermal parameters, and included in the structure factor calculations. The hydrogen atoms attached to oxygen were refined with O-H=0.85Å and U_iso_(H) = 1.2U_eq_(O). Crystallographic data for complexes **1**–**6** are given in Table 2. Selected bond lengths and angles for **1**–**6** are listed in Table S1. CCDC reference numbers: 1858388 for L, 1858385 for **1**, 1858386 for **2**, 1858389 for **3**, 1858390 for **4**, 1858391 for **5**, and 1858387 for **6**. Topological analysis was performed by using TOPOS program [22,23,24].

## 4. Conclusions

In summary, based on the designed X-shaped ligand **L**, six CPs were obtained with the structures ranging from a 1D polymeric fishbone chain (compound **4**), 1D polymeric loop chain (compounds **3** and **5**), 2D 4-connected {4^4^·6^2^}-**sql** sheet (compound **6**), 2D 4-connected {4^3^·6^3^}sheet (compound **2**), to a 3D (3,4)-connected {6·8^2^}{6^4^·8^2^}-**tcj** net (compound **1**). Most importantly, it should be noted that the geometric topologies of these CPs are not only controlled by the metal ions, but also by the related counter anions. Therefore, this work provides more detailed information on the development of unique CP structures in the solid state.

## Figures and Tables

**Table 1:** Detailed comparisons of complexes **1**–**6**.

	Metal Salts	τ_4_ Parameter ^a^	Average M-C_central_ (Å)	M-L Motifs	Final Structure
**1**	AgCF_3_SO_3_	0.44 (7)	9.310	2D [Ag_2_L]_n_ sheet	3D (3,4)-connected {6·8^2^}{6^4^·8^2^}-**tcj** net
**2**	AgSbF_6_	0.49 (7)	9.568	2D [AgL]_n_ sheet	2D 4-connected {4^3^·6^3^} sheet
**3**	ZnI_2_	0.67 (0)	9.559	1D [Zn_2_L]_n_ loop chain	1D polymeric loop chain
**4**	CdI_2_	0.36 (0)	8.825	1D [Cd_2_L]_n_ fishbone chain	1D polymeric fishbone chain
**5**	HgI_2_	0.65 (5)	9.883	1D [Hg_2_L]_n_ loop chain	1D polymeric loop chain
**6**	HgCl_2_	0.78 (1)	9.587	2D [Hg_2_L]_n_ bilayer	2D 4-connected{4^4^·6^2^}-**sql** sheet

^a^ τ_4_ parameter of the central C atom in the {CM_4_} tetrahedron.

**Table 2:** Crystal data for **1**–**6**.

Complex	Ligand	1	2	3	4	5	6
Empirical formula	C_29_H_36_N_12_O_6_	C_34_H_43_Ag_2_F_6_N_13_O_13_S_2_	C_79_H_117_Ag_2_Sb_2_F_12_N_31_O_17_	C_58_H_94_I_8_N_24_O_23_Zn_4_	C_41_H_70_Cd_2_I_4_N_16_O_13_	C_41_H_60_Hg_2_I_4_N_16_O_8_	C_38_H_58_Cl_4_Hg_2_N_15_O_9_
Formula weight	648.70	1235.67	2460.28	2772.25	1727.53	1813.82	1411.97
Crystal system	Triclinic	Monoclinic	Triclinic	Tetragonal	Triclinic	Monoclinic	Monoclinic
Space group	*P-1*	*P*2_1_/c	*P-1*	*I4/m*	*P-1*	*C*2/*m*	*P*2_1_
*a* (Å)	8.7220(10)	18.667(3)	10.9801(8)	17.902(9)	11.842(16)	26.3943(10)	17.782(8)
*b* (Å)	14.1492(14)	29.678(5)	16.3332(12)	17.902(9)	16.48(2)	15.4834(6)	8.948(4)
*c* (Å)	14.7268(17)	8.6711(15)	17.1655(12)	15.312(16)	17.71(2)	16.8250(8)	19.152(9)
*α* (°)	64.258(3)	90	66.498(2)	90	101.147(17)	90	90
*β* (°)	77.246(3)	102.504(4)	81.929(2)	90.00	108.606(16)	123.338(2)	110.646(7)
*γ* (°)	72.356(6)	90	87.312(2)	90	92.633(18)	90	90
*V* (Å^3^)	1551.6(3)	4689.8(14)	2795.0(3)	4907(6)	3191(7)	5744.4(4)	2852(2)
*Z*	2	4	1	2	2	2	2
*D*_calcd_ (Mg/m^3^)	1.388	1.750	1.462	1.876	1.798	1.759	1.644
*μ* (mm^ÿ1^)	0.101	1.022	0.913	3.555	2.665	7.523	5.623
*F* (000)	684	2488	1248	2676	1680	2776	1382
*R*_int_	0.0570	0.1292	0.0799	0.0479	0.0486	0.0508	0.0831
Final *R* indices ^a^	0.0479 (0.1111)	0.0971 (0.1915)	0.0803 (0.2284)	0.1259 (0.2454)	0.0587 (0.1492)	0.0382 (0.1026)	0.0695 (0.1486)
*R* indices (all data) ^a^	0.0802 (0.1366)	0.1999 (0.2349)	0.1363 (0.2734)	0.1427 (0.2540)	0.1172 (0.1492)	0.0453 (0.1065)	0.1624 (0.1729)
Gof	1.025	1.072	1.048	1.007	1.069	1.042	1.036

^a^*R*_1_ = Σ||*F*_o_| − |*F*_c_||/Σ|*F*_o_|, *wR*_2_ = [Σ*w*(*F*_o_^2^ÿ*F*_c_^2^)^2^]/Σ*w*(*F*_o_^2^)^2^]^1/2^.

## Supplementary Materials

The following are available online, Scheme S1: The synthesis route of the X–shaped urea-based ligand, Figure S1: ^1^H-NMR spectrum of the ligand **L**, Figure S2: PXRD patterns of **1** (a), **2** (b), **3** (c), **4** (d), **5** (e) and **6** (f), Figure S3: The IR spectra of the Ligand and Complexes **1**–**6**, Table S1: Selected bondlengths (Å) andangles (°C) for **1**–**6**.
