# Supplementary material for: Structural Diversity of Six Coordination Polymers Based on the Designed X-Shaped Ligand 1,1,1,1-Tetrakis[(3-pyridiniourea)methyl]methane
Source: Molecules. 2018 Sep 7;23(9):2292. doi: 10.3390/molecules23092292 (PMC6225222; doi:10.3390/molecules23092292)
Supplement: Supplementary file 1 [file molecules-23-02292-s001.pdf]

Supporting Information

Structural Diversity of Six Coordination Polymers Based on the Designed X-Shaped Ligand 1,1,1,1-Tetrakis[(3-pyridiniourea)methyl]methane

Qi-Long Zhang [1,2,*], Qing Yu [2], Hai-Fang Xie [2], Bo Tu [2,*], Hong Xu [2], Ya-Li Huang [2], Xiao-Sheng Yang [1]

[1] State Key Laboratory of Functions and Applications of Medicinal Plants, Guizhou Medical University, Guiyang 550014, China; gzcnp@sina.cn

[2] School of Basic Medical Science, Guizhou Medical University, Guiyang 550004, China; yq518328@126.com (Q.Y.); x1352316768@126.com (H.F.X.); xuhong@gmc.edu.cn (H.X.); ylh6401@gmc.edu.cn (Y.L.H.)

* Correspondence: sciqlzhang@gmc.edu.cn or gzuqlzhang@126.com (Q.L.Z.); tubo@gmc.edu.cn (B.T.); Fax: +86-851-8817-4017 (Q.L.Z.); +86-851-8817-4017 (B.T.)

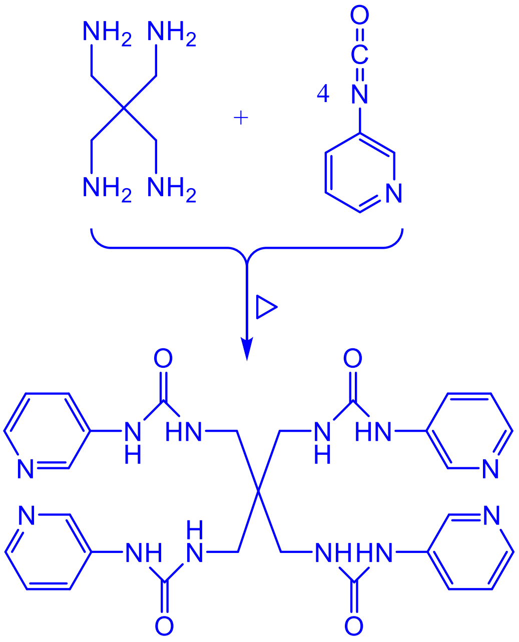

Scheme S1. The synthesis route of the X–shaped urea-based ligand.

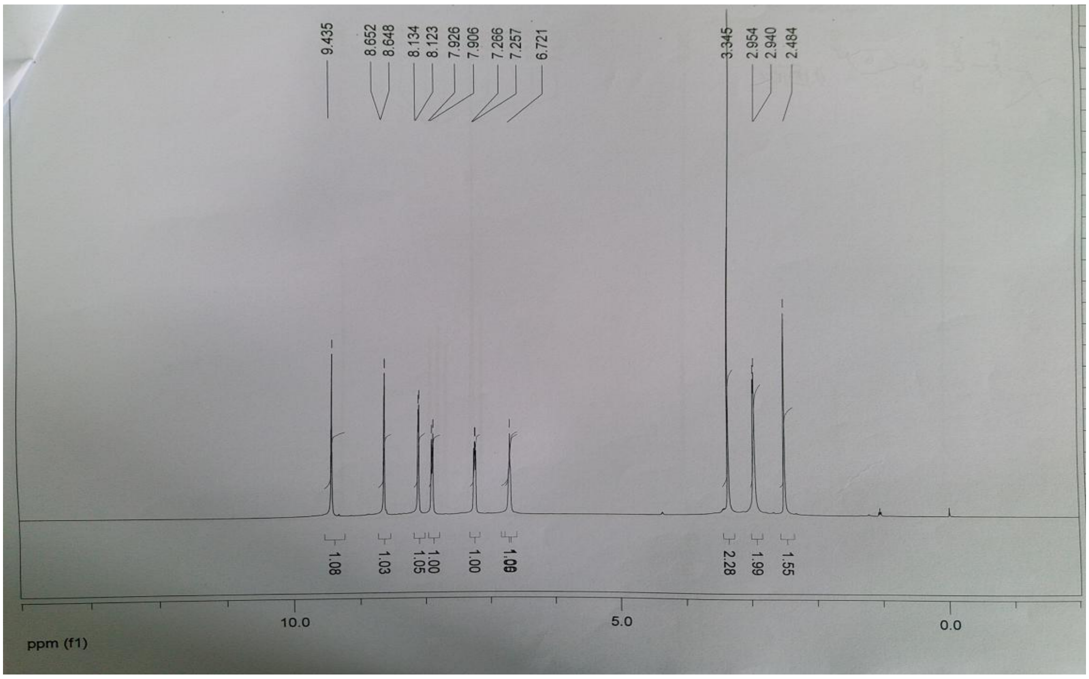

Figure S1. ^{1}H-NMR spectrum of the ligand **L**

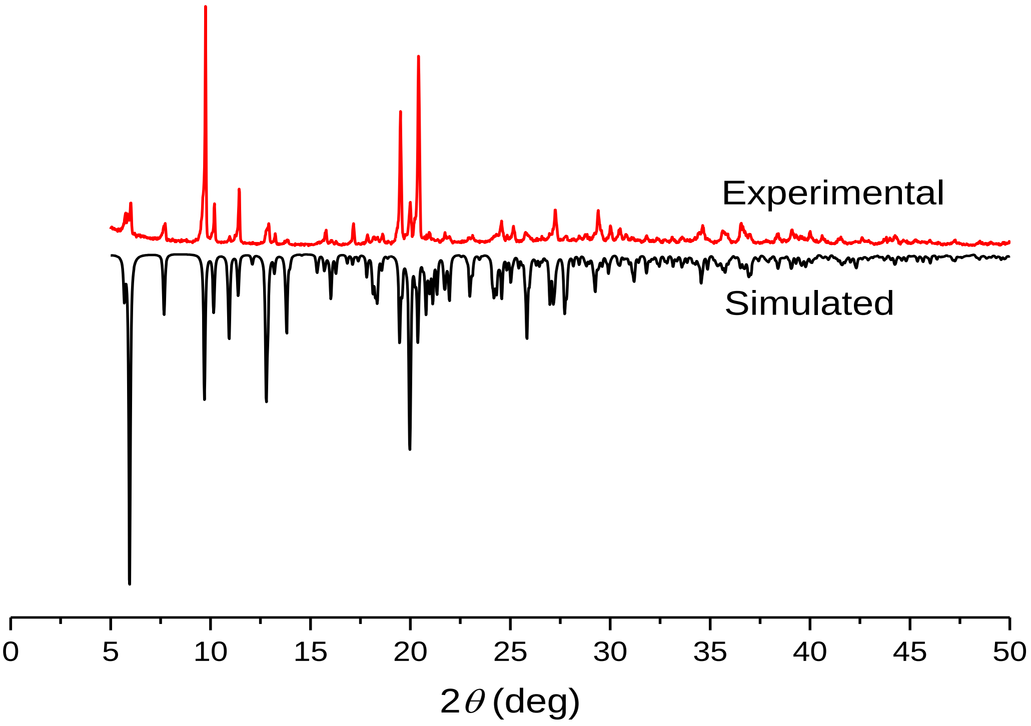

(a)

Figure S2. *Cont.*

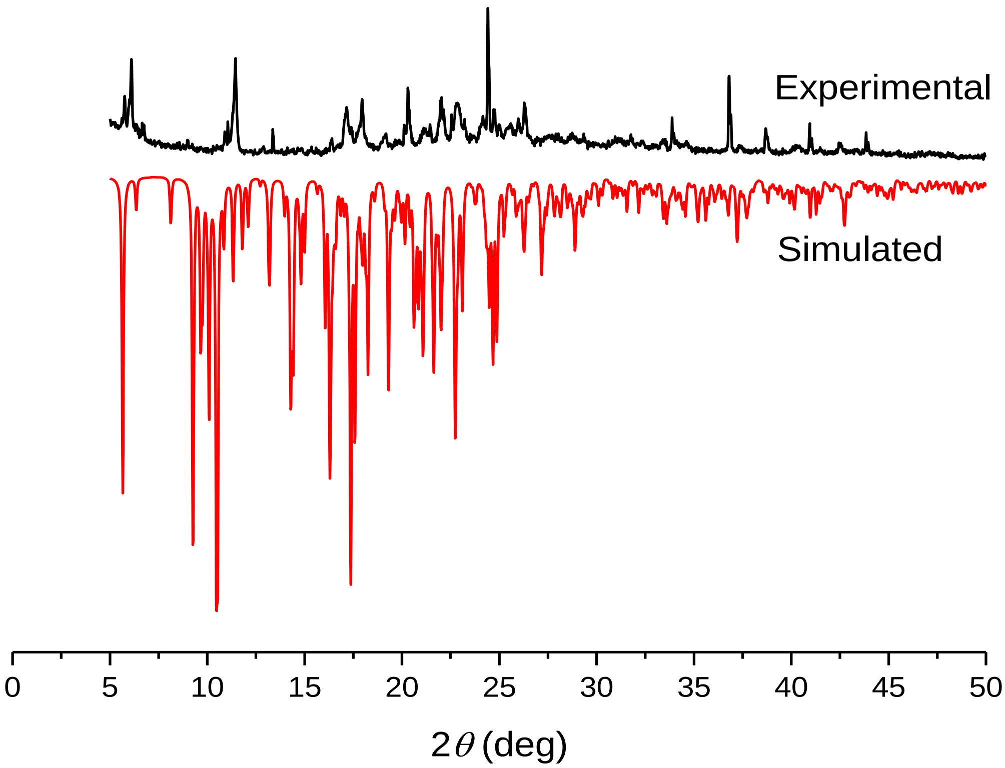

(b)

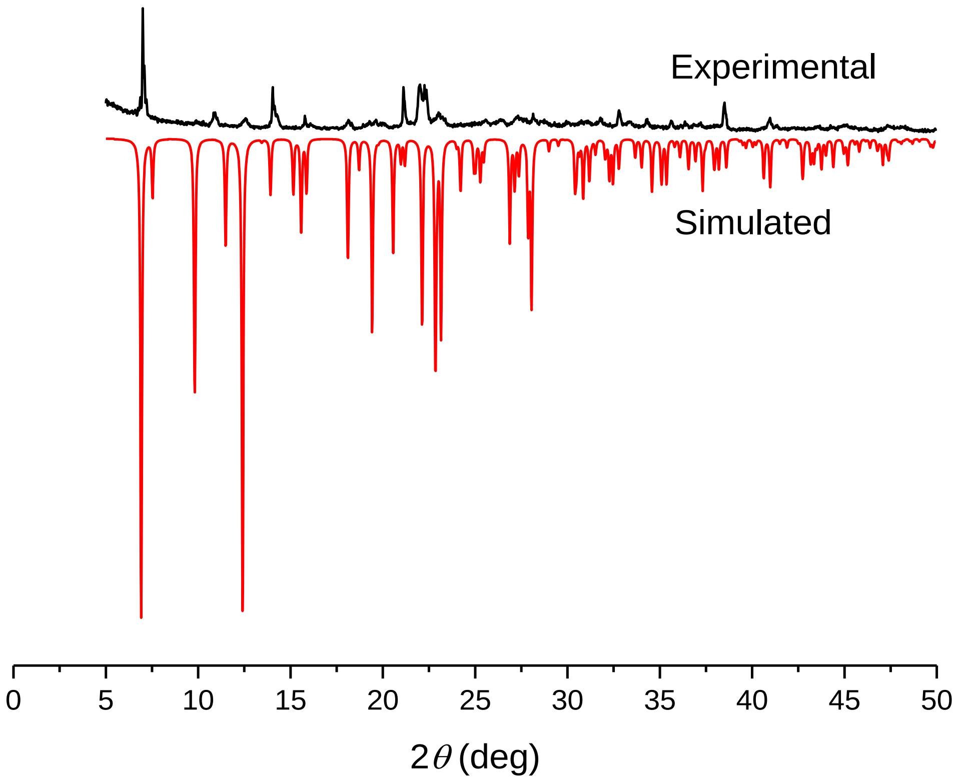

(c)

Figure S2. *Cont.*

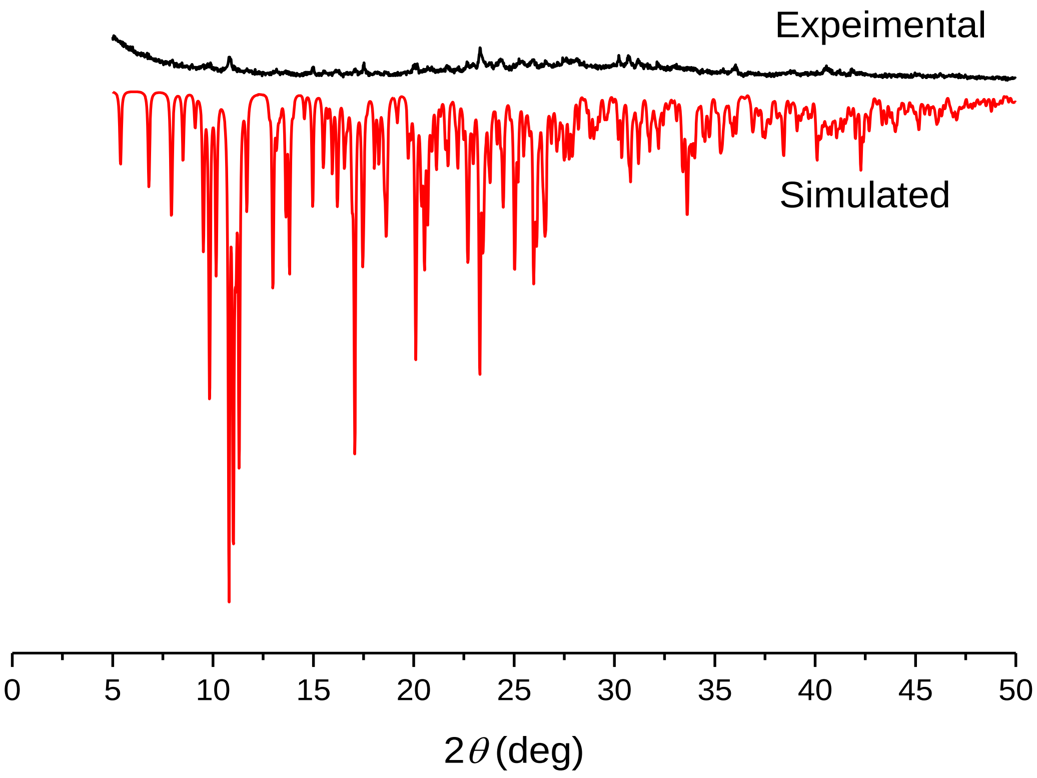

(d)

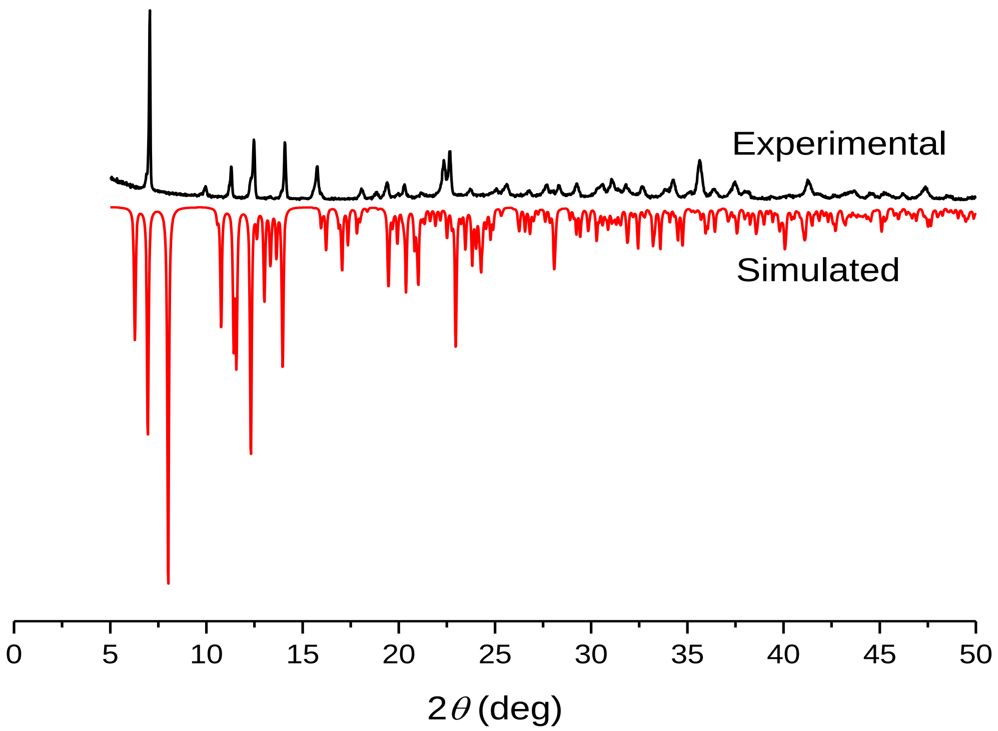

(e)

Figure S2. *Cont.*

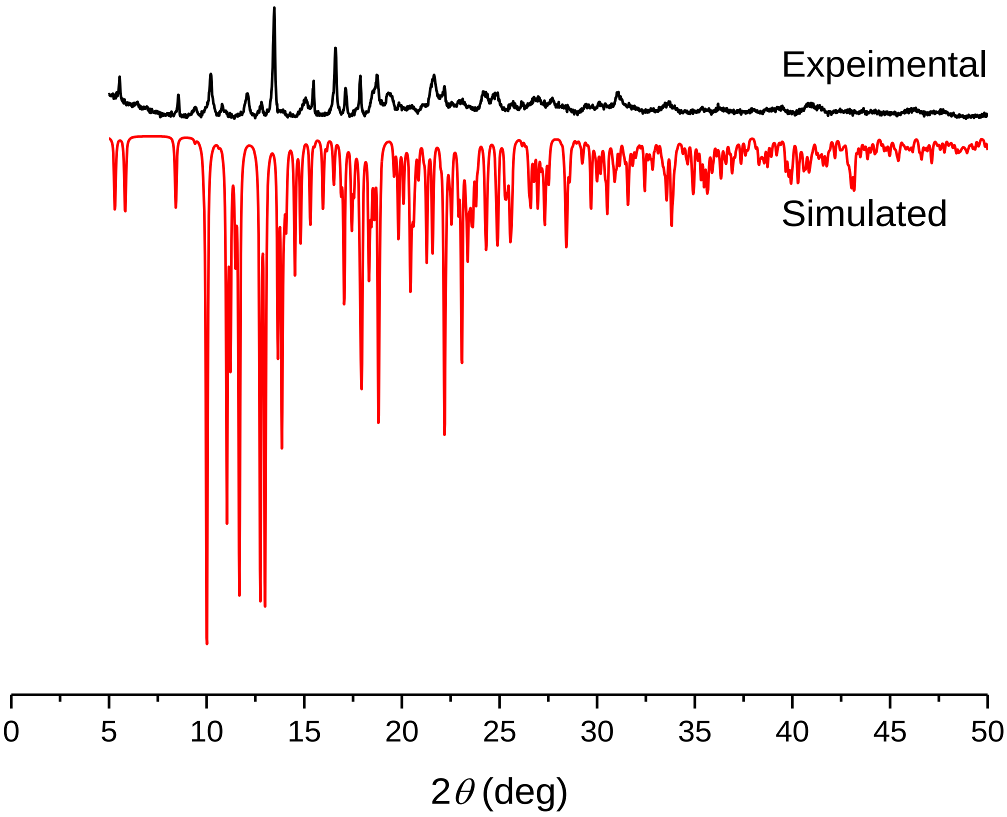

(f)

Figure S2. PXRD patterns of **1** (a), **2** (b), **3** (c), **4** (d), **5** (e) and **6** (f)

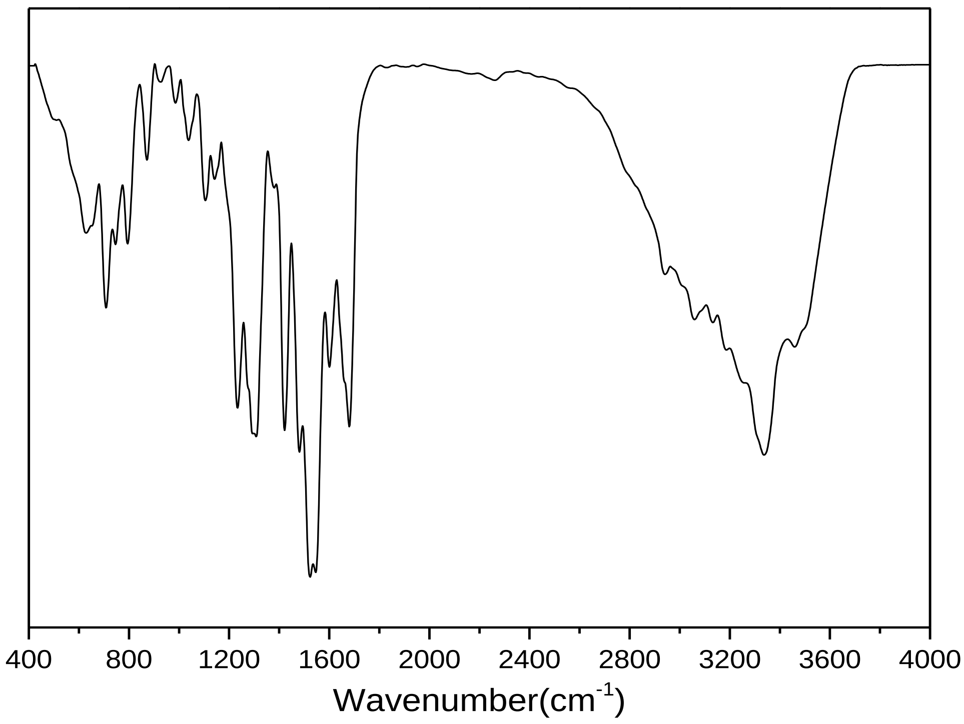

Ligand

Figure S3. *Cont.*

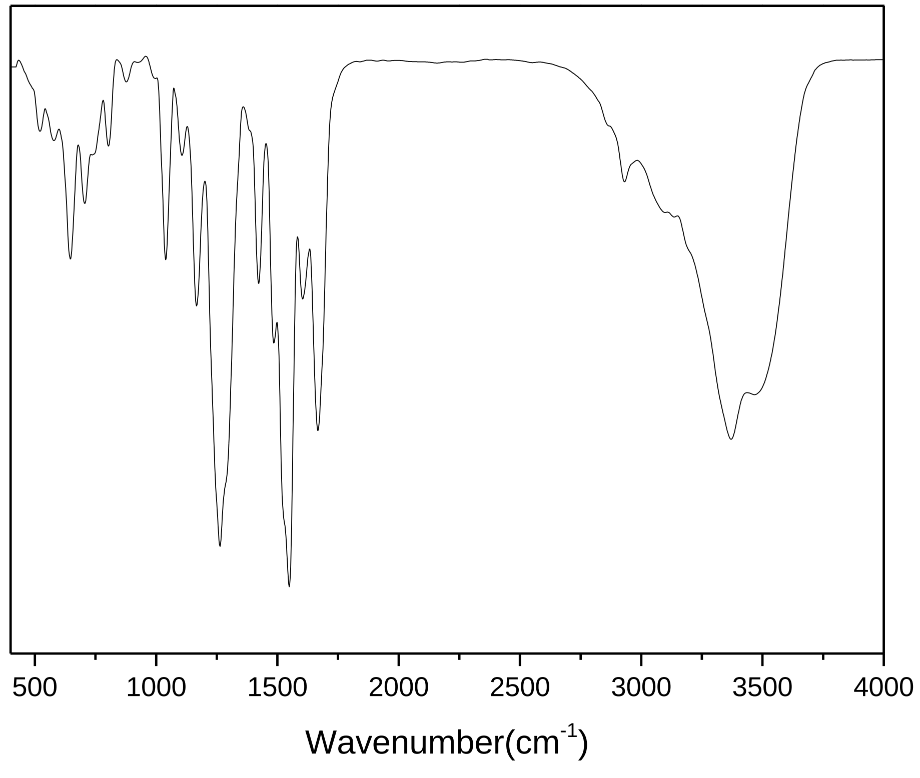

Complex **1**

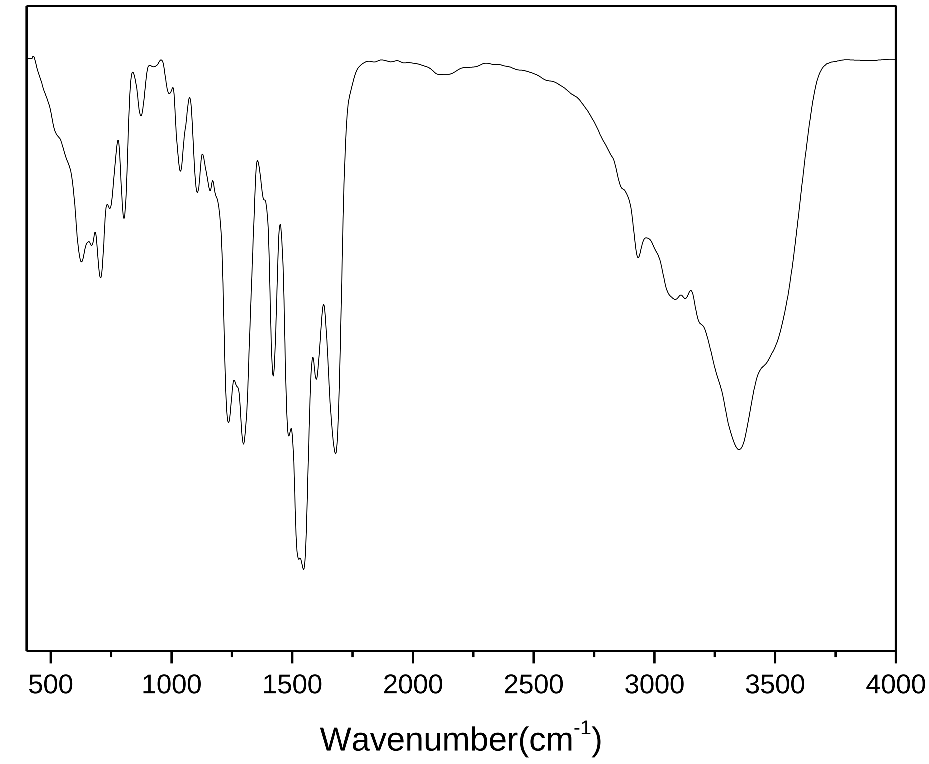

Complex **2**

Figure S3. *Cont.*

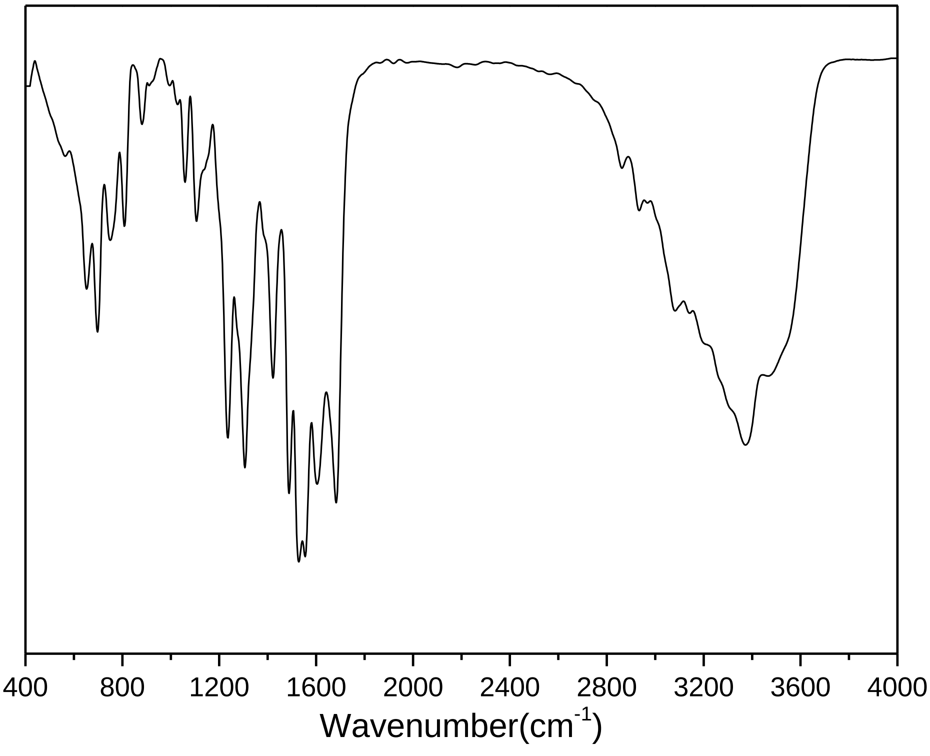

Complex **3**

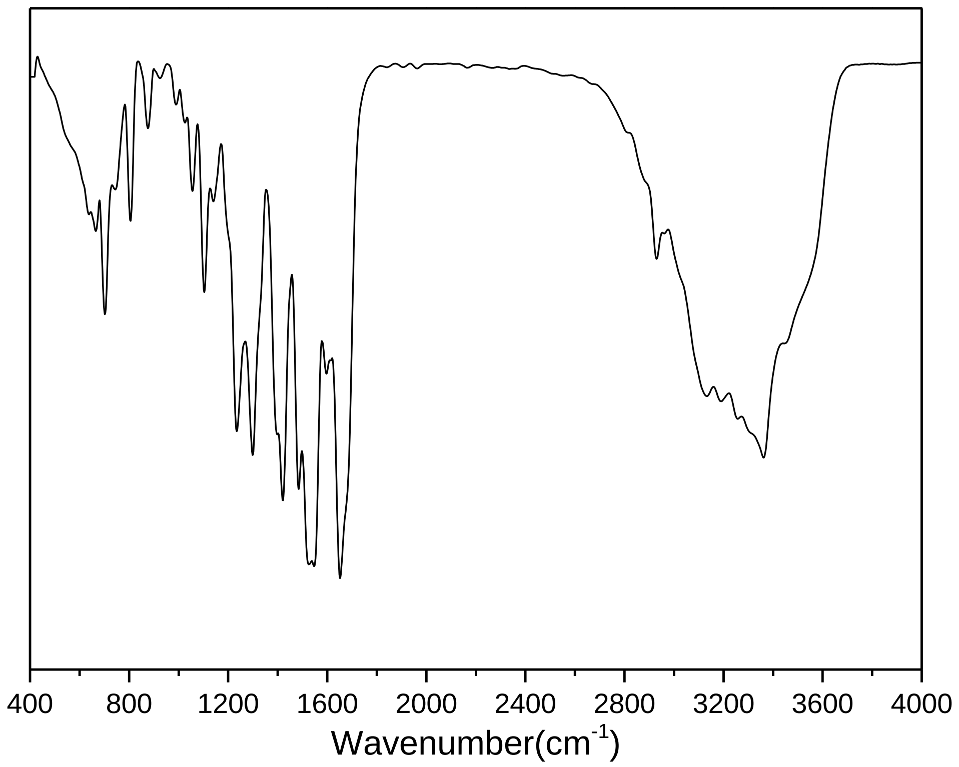

Complex **4**

Figure S3. *Cont.*

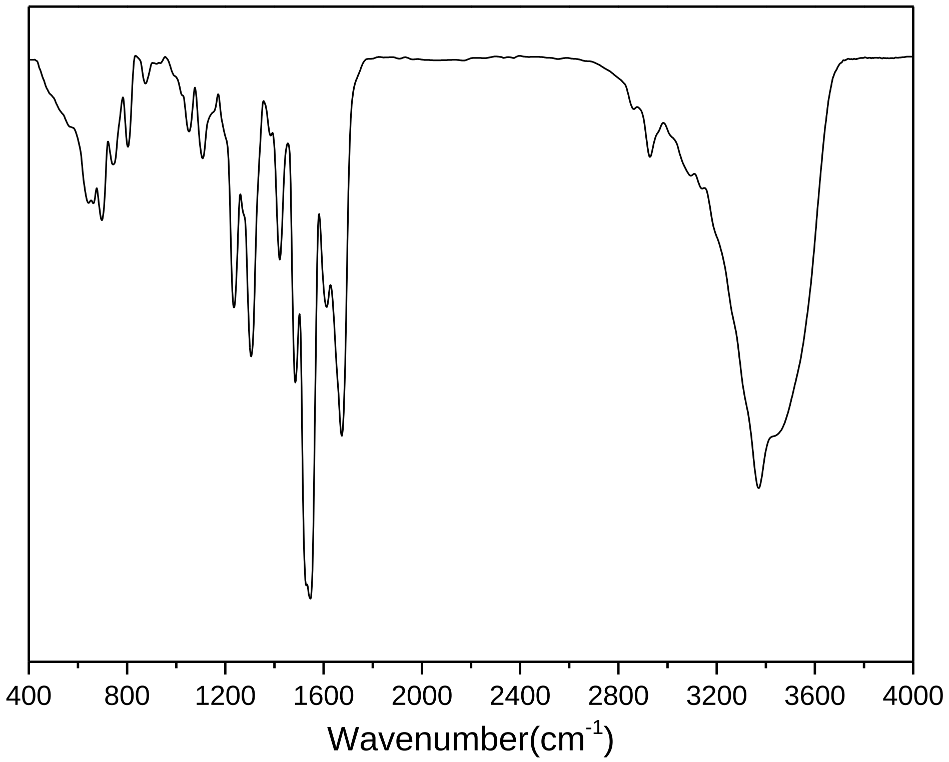

Complex **5**

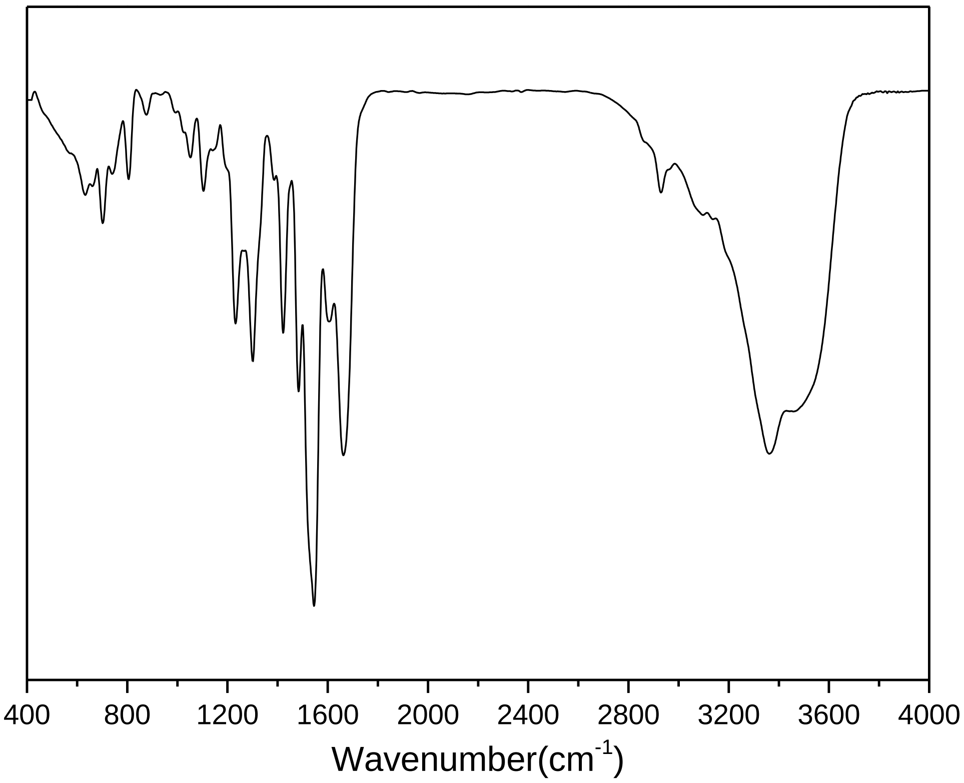

Complex **6**

Figure S3. The IR spectra of the Ligand and Complexes **1-6.**

Table S1.Selected bondlengths(Å)andangles(℃) for **1–6**.

**Complex 1**							
Ag2-N11	2.109(9)	Ag2-N12	2.118(8)	O9-Ag1	2.54(2)	N9-Ag1	2.131(7)
N10-Ag1	2.132(7)	N9-Ag1-N10	178.4(3)	N9-Ag1-O9	84.5(6)	N10-Ag1-O9	97.0(6)
N11-Ag2-N12	165.1(3)	N11-Ag2-Ag2	90.6(2)				
**Complex 2**							
N1-Ag1	2.239(7)	N9-Ag1	2.357(7)	N12-Ag1	2.415(7)	N6-Ag1	2.303(7)
N1-Ag1-N9	112.9(2)	N1-Ag1-N12	111.3(3)	N1-Ag1-N6	131.5(2)	N9-Ag1-N12	94.9(2)
N6-Ag1-N9	102.8(2)	N6-Ag1-N12	96.6(3)				
Complex 3							
Zn1-N1	2.020(6)	Zn1-I2	2.577(3)	Zn1-I1	2.637(4)	Zn1A-N1	2.013(7)
Zn1A-I2A	2.586(4)	Zn1A-I1A	2.645(10)	N1-Zn1-N1	105.7(4)	N1-Zn1-I2	112.99(18)
N1-Zn1-I2	112.99(18)	N1-Zn1-I1	100.51(19)	N1-Zn1-I1	100.51(19)	I2-Zn1-I1	122.16(12)
N1-Zn1A -N1	106.1(4)	N1-Zn1A-I2A	103.86(19)	N1-Zn1A-I2A	103.86(19)	N1-Zn1A-I1A	88.3(2)
N1-Zn1A-I1A	88.3(2)	I2A-Zn1A-I1A	159.4(2)				
**Complex 4**							
O1W-Cd1	2.407(9)	O5-Cd1	2.338(7)	Cd1-N1	2.327(8)	Cd1-N4	2.364(8)
Cd1-N10	2.376(8)	Cd1-I4	2.855(4)	Cd2-N7	2.339(8)	Cd2-I2	2.728(3)

Cd2-I3	2.733(2)	Cd2-I1	2.743(3)	N1-Cd1-O5	88.0(3)	N1-Cd1-N4	95.3(3)
O5-Cd1-N4	165.1(3)	N1-Cd1-N10	173.3(3)	I3-Cd2-I1	117.39(10)	I2-Cd2-I1	112.42(9)
O5-Cd1-N10	86.8(3)	N4-Cd1-N10	88.7(3)	N1-Cd1-O1W	86.8(3)	O5-Cd1-O1W	81.2(3)
N4-Cd1-O1W	84.5(3)	N10-Cd1-O1W	88.2(3)	N1-Cd1-I4	91.8(2)	O5-Cd1-I4	98.2(2)
N4-Cd1-I4	96.2(2)	N10-Cd1-I4	93.1(2)	O1W-Cd1-I4	178.5(2)	N7-Cd2-I2	103.2(2)
N7-Cd2-I3	102.0(3)	I2-Cd2-I3	118.40(9)	N7-Cd2-I1	99.5(2)		
**Complex 5**							
N1-Hg1	2.452(6)	N6-Hg2	2.394(5)	I1-Hg1	2.6434(9)	I2-Hg1	2.6416(7)
I3-Hg2	2.6511(8)	I4-Hg2	2.6499(9)	Hg1-N1	2.452(6)	Hg2-N6	2.394(5)
N1-Hg1-N1	91.1(3)	N1-Hg1-I2	99.29(14)	N1-Hg1-I2	99.29(14)	N1-Hg1-I1	100.55(15)
N1-Hg1-I1	100.55(15)	I2-Hg1-I1	151.51(3)	N6-Hg2-N6	85.6(3)	N6-Hg2-I4	104.79(13)
N6-Hg2-I4	104.79(13)	N6-Hg2-I3	103.79(13)	N6-Hg2-I3	103.79(13)	I4-Hg2-I3	140.66(3)
**Complex 6**							
N1-Hg1	2.398(9)	N4-Hg1	2.449(11)	N7-Hg2	2.424(8)	N10-Hg2	2.415(11)
Cl1-Hg1	2.314(3)	Cl2-Hg1	2.330(4)	Cl3-Hg2	2.329(6)	Cl4-Hg2	2.325(5)
Cl1-Hg1-Cl2	152.97(15)	Cl1-Hg1-N1	100.8(2)	Cl2-Hg1-N1	103.2(2)	Cl1-Hg1-N4	95.4(3)
Cl2-Hg1-N4	99.6(3)	N1-Hg1-	N4 83.1(4)	Cl4-Hg2-Cl3	146.4(2)	Cl4-Hg2-N10	101.2(3)
Cl3-Hg2-N10	101.5(3)	Cl4-Hg2-N7	102.6(3)	Cl3-Hg2-N7	100.7(3)	N10-Hg2-N7	92.4(3)
